# APE1 redox function is required for activation of Yes-associated protein 1 under reflux conditions in Barrett’s-associated esophageal adenocarcinomas

**DOI:** 10.1186/s13046-022-02472-5

**Published:** 2022-09-01

**Authors:** Farah Ballout, Heng Lu, Lei Chen, Kannappan Sriramajayam, Jianwen Que, Zhipeng Meng, Timothy C. Wang, Silvia Giordano, Alexander Zaika, Oliver McDonald, Dunfa Peng, Wael El-Rifai

**Affiliations:** 1 Department of Surgery, Miller School of Medicine, University of Miami, Rosenstiel Med Science Bldg., 1600 NW 10th Ave, Room 4007, Miami, FL 33136-1015 USA; 2 Department of Gastroenterology, Beijing Friendship Hospital, Capital Medical University, Beijing, P. R. China; 3 Department of Medicine, Columbia University, New York, NY 10027 USA; 4 Department of Molecular and Cellular Pharmacology & Sylvester Comprehensive Cancer Center, University of Miami Miller School of Medicine, Miami, FL USA; 5 Department of Medicine and Herbert Irving Comprehensive Cancer Center, Columbia University Irving Medical Center, New York, NY USA; 6 Department of Oncology, University of Torino and Candiolo Cancer Institute, FPO-IRCCS, 10060 Candiolo, Italy; 7 Department of Veterans Affairs, Miami Healthcare System, Miami, FL USA; 8 Department of Pathology, Miller School of Medicine, University of Miami, Miami, FL 33136 USA; 9 Sylvester Comprehensive Cancer Center, Miller School of Medicine, University of Miami, Miami, FL USA

**Keywords:** Barrett’s esophagus, Reflux, Stemness, Redox, Cancer

## Abstract

**Background:**

Esophageal adenocarcinoma (EAC) is characterized by poor prognosis and low survival rate. Chronic gastroesophageal reflux disease (GERD) is the main risk factor for the development of Barrett’s esophagus (BE), a preneoplastic metaplastic condition, and its progression to EAC. Yes-associated protein 1 (YAP1) activation mediates stem-like properties under cellular stress. The role of acidic bile salts (ABS) in promoting YAP1 activation under reflux conditions remains unexplored.

**Methods:**

A combination of EAC cell lines, transgenic mice, and patient-derived xenografts were utilized in this study. mRNA expression and protein levels of APE1 and YAP1 were evaluated by qRT-PCR, western blot, and immunohistochemistry. YAP1 activation was confirmed by immunofluorescence staining and luciferase transcriptional activity reporter assay. The functional role and mechanism of regulation of YAP1 by APE1 was determined by sphere formation assay, siRNA mediated knockdown, redox-specific inhibition, and co-immunoprecipitation assays.

**Results:**

We showed that YAP1 signaling is activated in BE and EAC cells following exposure to ABS, the mimicry of reflux conditions in patients with GERD. This induction was consistent with APE1 upregulation in response to ABS. YAP1 activation was confirmed by its nuclear accumulation with corresponding up-regulation of YAP1 target genes. APE1 silencing inhibited YAP1 protein induction and reduced its nuclear expression and transcriptional activity, following ABS treatment. Further investigation revealed that APE1-redox-specific inhibition (E3330) or APE1 redox-deficient mutant (C65A) abrogated ABS-mediated YAP1 activation, indicating an APE1 redox-dependent mechanism. APE1 silencing or E3330 treatment reduced YAP1 protein levels and diminished the number and size of EAC spheroids. Mechanistically, we demonstrated that APE1 regulated YAP1 stability through interaction with β-TrCP ubiquitinase, whereas APE1-redox-specific inhibition induced YAP1 poly-ubiquitination promoting its degradation.

**Conclusion:**

Our findings established a novel function of APE1 in EAC progression elucidating druggable molecular vulnerabilities via targeting APE1 or YAP1 for the treatment of EAC.

**Supplementary Information:**

The online version contains supplementary material available at 10.1186/s13046-022-02472-5.

## Background

The incidence of esophageal adenocarcinoma (EAC) has been increasing in the United States over the past several decades [1]. Barrett esophagus (BE), a pre-malignant condition characterized by conversion of normal stratified esophageal squamous epithelium into metaplastic columnar epithelium, is the main risk factor for EAC. Chronic gastroesophageal reflux disease (GERD), where bile acids abnormally refluxate into the lower esophagus, induces high levels of oxidative stress with activation of oncogenic signaling pathways leading to the development of BE [2] and its progression to EAC [3–5].

Apurinic/apyrimidinic endonuclease 1 (APE1) plays a crucial role in the base excision repair (BER) pathway [6]. APE1 also exerts non-repair functions including a unique redox regulation of transcription factors. APE1 interacts with various redox-dependent transcription factors such as NF-κB, STAT3, HIF1α, and TP53 and maintains the reduced status of their cysteine residues, thus enhancing binding affinity on target genes [7, 8]. This multifunctionality is associated with APE1’s structure characterized by three functional domains: the N-terminal region devoted to redox-mediated transcriptional co-activation activity, the central region that exerts non-repair functions and the C-terminal domain responsible for DNA repair activity [9, 10]. APE1 has been shown to be aberrantly overexpressed in several tumors including ovarian, lung, liver and EAC [4, 11–13] and its overexpression has been associated with increased cell proliferation, invasion, metastasis, and drug resistance [14]. APE1 is upregulated in EAC cells, following exposure to acidic bile salts (ABS) [12, 15].

YES-associated protein 1 (YAP1) is a transcriptional co-activator that interacts with several transcription factors, most notably the family of TEAD transcription factors (TEAD1-4) [16]. YAP1 is the principal downstream effector of the Hippo signaling pathway. It is negatively regulated by upstream Hippo pathway core kinases including Mst1/2 and Lats1/2 resulting in its exclusion from the nucleus or its proteasomal degradation [17]. YAP1 plays an important role in normal tissue development, homeostasis, and regeneration [18]. A high YAP1 activity has been associated with proliferation, survival, stemness, invasion, and therapy resistance in various cancers [19, 20]. YAP1 overexpression has been described in high-grade dysplasia and EAC [21]. Increased YAP1 expression in esophageal cancer cells was associated with the acquisition of cancer stem cell (CSC)-like properties [22, 23]. The increase in YAP1 activity can induce EGFR expression in esophageal cancer cells, thus conferring resistance to 5-fluorouracil and docetaxel [24].

Although earlier studies imply activation of APE1 and YAP1 in esophageal cancer; the mechanistic relationship between these two proteins and their regulation in response to reflux conditions in EAC remains undetermined. In this study, we demonstrated that APE1 is a positive upstream regulator of YAP1 and promotes its upregulation following exposure to acidic bile salts, the mimic of reflux conditions in GERD patients. Activation of YAP1 signaling was dependent on APE1 redox function. APE1 enhanced YAP1 protein stability via interaction with β-TrCP ubiquitinase thus inhibiting YAP1 ubiquitination and degradation. These findings uncover a novel mechanistic link between APE1 and YAP1 in response to reflux conditions, the main risk factor for BE and EAC.

## Methods

### Cell Culture

FLO1 and OE33 cell lines were a kind gift from Dr. David Beer (University of Michigan, Ann Arbor, MI). SK-GT-4 cell line was kindly provided from Dr. Xiaochun Xu (MD Anderson Cancer Center, Houston, TX). Human CP-B cells were obtained from ATCC. OE33 cells were maintained in RPMI 1640 media (GIBCO), supplemented with 10% fetal bovine serum (FBS) (GIBCO), and 1% penicillin/streptomycin (GIBCO). FLO-1 and SK-GT-4 cells were maintained in DMEM media (GIBCO) containing 10% FBS and 1% penicillin/streptomycin. CP-B cells were maintained in DMEM-F12 media (GIBCO) containing 5% FBS, 1% penicillin/streptomycin, bovine pituitary extract, hydrocortisone, recombinant epidermal growth factor, 1X insulin transferrin selenium supplement and L-adenine. All cells were grown at 370C in a humidified incubator with 5% CO2. All cell lines were regularly authenticated and tested for mycoplasma contamination, using mycoplasma detection Kit (PCR) purchased from SouthernBiotech.

### Antibodies and reagents

A list of all primary and secondary antibodies used in this study is provided in Table 1.

**Table 1:** Primary and secondary antibodies used in this study

**Antibody**	**Source**	**Catalog#**	**Species**	**Dilution**	**Application**^a^
YAP	Cell signaling	14074	Rabbit	1:1000 1:200	WB IHC
YAP	Santa Cruz	sc-101199	Mouse	1:100	IF
APE1	Invitrogen	MA1-440	Mouse	1:7000	WB
APE1	Invitrogen	PA5-29157	Rabbit	1:200	IF
β-actin	Sigma Aldrich	A5441	Mouse	1:10,000	WB
P84	Genetex	GTX70220	Mouse	1:1000	WB
β-tubulin	Cell signaling	2128	Rabbit	1:1000	WB
β-TrCP	Invitrogen	37–3400	Mouse	1:100	PLA
β-TrCP	Cell signaling	4394	Rabbit	1:1000	WB
Flag antibody	Sigma Aldrich	F1804	Mouse	2 µg	IP
Ubiquitin	Cell signaling	43124	Rabbit	1:1000	WB
Anti-rabbit IgG HRP-linked Antibody	Cell signaling	7074	Goat	1:5000	WB
Anti-mouse IgG HRP-linked Antibody	Cell signaling	7076	Goat	1:5000	WB
Alexa Fluor 488 goat anti-mouse IgG	Invitrogen	A-11029	Goat	1:200	IF
Alexa Fluor 568 goat anti-rabbit IgG	Invitrogen	A-11011	Goat	1:200	IF
Mouse Anti-Rabbit IgG (Light-Chain Specific) HRP Conjugate	Cell signaling	93702	Mouse	1:1000	WB
Goat Anti-Mouse IgG (Light-Chain Specific) HRP Conjugate	Cell signaling	91196	Goat	1:1000	WB

^a^*IF* Immunofluorescence, *WB* Western blot, *IP* Immunoprecipitation, *IHC* Immunohistochemistry, *PLA* Proximity ligation assay

The APE1 redox-specific inhibitor E3330 was purchased from Novus Biologicals (NBP1-49,581, Centennial, CO, USA).

### Transfection and lentivirus infection

Scrambled si-RNA (sc-29470) was purchased from Santa Cruz Biotechnology. APE1 si-RNA (L-010237–00-0005) was obtained from Dharmacon (Lafayette, CO, USA). The flag-tagged coding sequence of APE1 was cloned in pcDNA3.1 mammalian expression plasmid (Invitrogen, Carlsbad, CA, USA). APE1 redox-deficient mutant (C65A) was developed by QuickChange Lightning Site-Directed Mutagenesis Kit (Agilent Technologies, Santa Clara, CA, USA). The flag-tagged coding sequence of YAP1 was cloned in pcDNA3.1 mammalian expression plasmid (Invitrogen, Carlsbad, CA, USA). The mammalian expression HA-Ubiquitin plasmid was purchased from Addegene (Addgene plasmid # 18,712; http://n2t.net/addgene:18712; RRID: Addgene18712) [25]. For transient overexpression of APE1 or YAP1, mammalian expression plasmids or empty vector were transfected into OE33 and FLO-1 cells using PolyJet reagent (SignaGen Laboratories, Rockville, MD, USA). For transient knockdown of APE1, si-APE1 or scrambled si-RNA were transfected into OE33 and FLO-1 cells using LipoJet reagent (SignaGen Laboratories, Rockville, MD, USA). Cells were harvested within 72 h of transient transfection.

### Acidic bile salts treatment

Bile salts (BS) cocktail was prepared by mixing equal concentrations of glycocholic acid (GCA), taurocholic acid (TCA), glycodeoxycholic acid (GDCA), glycochenodeoxycholic acid (GCDCA) and deoxycholic acid (DCA). Cells were treated with 200 μM BS cocktail in corresponding acidic culture media (pH 4.0, ABS) for 20 min, followed by recovery in regular media.

### Cycloheximide chase assay

OE33 and FLO-1 cells were pre-treated with 100 μg/mL cycloheximide (CHX) for 2 h followed by exposure to ABS (pH 4, 200 μM) for 20 min before lysates were collected at different time points and analyzed for APE1 and YAP1 protein levels by Western blot. OE33-scramble and OE33-si-APE1 cells were treated with 100 μg/mL CHX and whole cell lysates were collected at different time points and analyzed by Western blot.

### Sphere formation assay

Single-cell suspension of OE33 cells was counted, and a density of 2000 cells/well was suspended in cold Growth Factor Reduced Matrigel™/ serum-free RPMI-1640 medium (1:1) in a total volume of 50 µl [26]. Each experimental condition was performed in triplicate. The master mix of cells with Matrigel™ was plated at the rim of the well of a 24-well plate and allowed to solidify in the incubator at 37 °C for 45 min. RPMI media containing 5% FBS (with or without treatment) was added gently at the center of the well. Media or treatments were replenished every two days. Sphere counts and imaging were performed at day 10 of sphere culture.

### Western blot analysis

Cells were lysed in RIPA lysis buffer (sc-24948, Santa Cruz, CA, USA). Protein extracts were quantified using the DC Bio-Rad Protein Assay (Bio-Rad Laboratories, Hercules, California, USA) according to the manufacturer’s protocol. Protein samples were mixed with 4X Laemmli sample buffer and heated at 85 °C for 10 min for gel electrophoresis. An equal amount of protein lysate was separated on 10% SDS–PAGE for 90 min at 100 V then transferred onto nitrocellulose membrane (Bio-Rad, CA, USA). Membranes were blocked with 5% BSA for 1 h and then incubated overnight at 4 °C with the primary antibody (refer to Table 1 for antibody source and dilution). Membranes were then washed three times with 1X TBST and incubated with the corresponding secondary antibody for 1 h at room temperature. Target proteins were detected using commercial Immobilon Western Chemiluminescent HRP Substrate detection reagents (ThermoFisher Scientific). Images were generated and quantified using ChemiDoc™ Imaging Systems (Bio-Rad, CA, USA).

### Immunoprecipitation

Cells were lysed in Pierce™ IP Lysis Buffer (ThermoFisher Scientific) supplemented with 1 × Halt protease inhibitor cocktail and 1 × Halt phosphatase inhibitor cocktail (ThermoFisher Scientific). Cell lysates were rotated together with Protein G Magnetic beads and 2ug of antibody or control IgG at 4 °C overnight. The immunocomplexes were then washed eight times with PBST buffer (PBS containing 0.1% Tween® 20) and subject to western blot analysis as described previously.

### Ubiquitination Assay for Mammalian Cells

Cells were co-transfected with HA-Ubiquitin plasmid and Flag YAP1 using Polyjet. Cells were then treated with 10 µM MG132 (Sigma-Aldrich) for 6 h and harvested within 72 h of transfection. Cells were then lysed and immunoprecipitated with Flag antibody (Sigma-Aldrich) as mentioned before. Samples were then subject to western blot analysis with anti-ubiquitin antibody (Cell signaling) to determine the primary proteins’ ubiquitination levels.

### Proximity ligation assay

In situ protein–protein interactions were detected using the Duolink in situ proximity ligation assay (PLA) detection kit (Sigma-Aldrich) following the manufacturer’s instructions. Cells were cultured in 8-well chamber slides for 24 h and then washed with PBS and fixed with 4% paraformaldehyde for 45 min at room temperature. Cells were then permeabilized with 0.5% Triton X-100 in PBS for 10 min, blocked for 1 h in a humidity chamber at 37 °C, and incubated overnight at 4 °C with the two primary antibodies raised against the two proteins of interest, each from a different host species. The primary antibodies (APE1, Rabbit monoclonal, ThermoFisher Scientific; β-TrCP, Mouse Monoclonal, ThermoFisher Scientific) were used. Hybridization, ligation, washing, and detection steps were performed following the supplier’s protocol. After final washes in buffer B, cells were mounted using the Duolink in situ mounting medium with DAPI, sealed with nail polish, and allowed to dry for 15 min at room temperature before imaging using the All-in-One Fluorescence Microscope (BZ-X700, Keyence Corp, Atlanta, GA).

### Cell fractionation

Cells were transfected with scramble or si-APE1 then treated with 200 μM ABS for 20 min followed by recovery in regular media at different time points. Cells were trypsinized and processed for cytosol and nuclear separation following the manufacturer’s protocol (ThermoFisher Scientific, USA). Briefly, cells were trypsinized, washed with 1X PBS, and centrifuged at 500 g for 5 min. The pellet was then resuspended in cytoplasmic extraction reagent I (CER I), vortexed for 15 s and incubated on ice for 10 min. Cytoplasmic extraction reagent II (CER II) was then added, followed by vortexing and centrifugation at 16,000 g for 5 min. The supernatant was collected as the cytosolic fraction. The pellet was resuspended nuclear extraction reagent (NER), vortexed for 15 s, incubated on ice for 40 min with vortexing every 10 min, and centrifuged at 16,000 g for 10 min. The resulting supernatant was collected as the nuclear fraction. The isolated cytosolic and nuclear fractions were mixed with the 4X Laemmli sample buffer and electrophoresed using SDS-PAGE.

### Quantitative real-time RT-PCR

Total RNA was extracted using Direct-zol RNA Miniprep Plus Kit according to manufacturer’s protocol. Total 1 μg/sample RNA was subjected to cDNA synthesis using the iScript™ cDNA Synthesis Kit (Biorad). The primers used were designed using primer 3 online tools (http://bioinfo.ut.ee/primer3-0.4.0/primer3/) and were obtained from Integrated DNA Technologies (Coralville, Iowa). Quantitative real-time polymerase chain reaction (qRT-PCR) was carried out using an iCycler (Biorad laboratories). All reactions were performed in triplicates. The fold expression was calculated and normalized to the average CT value of HPRT housekeeping gene. The real-time PCR primers were purchased from Integrated DNA Technologies (IDT, Coralville, IA). Primer sequences were as follows: APE1 (Forward: GCTTCGAGCCTGGATTAAGA; Reverse: TTGGTCTCTTGAAGGCACAGT), YAP1 (Forward: GATGAACTCGGCTTCAGGTC; Reverse: TTGGGTCTAGCCAAGAGGTG), CTGF (Forward: TGGAGATTTTGGGAGTACGG; Reverse: CAGGCTAGAGAAGCAGAGCC), CYR61 (Forward: CCCGTTTTGGTAGATTCTGG; Reverse: GCTGGAATGCAACTTCGG), ANKRD1 (Forward: AGCCCTCATGCTTGCTGTAT; Reverse: TTTGTTCATGAATGTGATGAAATC), HPRT (Forward: ACCCTTTCCAAATCCTCAGC; Reverse: GTTATGGCGACCCGCAG).

### Luciferase reporter assay

Cells were transfected with scramble or si-APE1 using lipojet transfecting agent. The next day cells were co-transfected with 8xGTIIC-luciferase plasmid (Addgene plasmid # 34,615; http://n2t.net/addgene:34615; RRID: Addgene_34615) [27], as a measure of YAP/TEAD transcription activity, along with renilla as the internal control using polyjet DNA transfecting agent. 48 h after transfection, cells were treated with a 200 μM mixture of acidic bile salts (pH4) for 20 min and allowed to recover for 3 h in complete medium. The cells were harvested and lysed with 1X luciferase passive lysis buffer. A FLUOstar OPTIMA microplate reader (BMG LABTECH, Cary, NC) was used to measure luciferase activity after adding the luciferase reagent and renilla after adding the stop solution using a dual-luciferase reporter assay system (Promega). Luciferase activity was calculated by normalizing the luciferase with the corresponding renilla value and reported as relative luciferase activity.

### Immunocytochemistry of embedded spheres

Paraffin-embedded sphere slides were deparaffinized and rehydrated following standard protocols. Antigen retrieval was performed by boiling the slides in 1 mmol/L Tris EDTA, pH 8.0 for 20 min. Slides were allowed to cool down to room temperature before incubation in 10% normal goat serum blocking solution (Thermo Fisher Scientific) for 1 h. Slides were incubated with primary antibodies overnight at 4 °C in a humidified chamber. Slides were washed with PBS and incubated with secondary antibody (Alexa Fluor 488 and Alexa Fluor 568) for 1 h at room temperature. Finally, slides were washed and mounted using mounting medium with DAPI (Abcam; ab104139). Fluorescent signals were captured using BZ-X710 KEYENCE All-in-One Fluorescence Microscope.

### Immunofluorescence staining

Cells were seeded in 6 well plates on coverslips. The cells were washed with PBS and treated with ABS (200 μM for 20 min), washed again with PBS, and allowed to recover for 3 h in complete medium. Cells were fixed with 4% paraformaldehyde for 45 min, and then permeabilized with 0.5% Triton X-100 for 10 min. The cells were then washed, blocked using goat antiserum for 20 min at room temperature and then incubated with primary antibody anti-YAP (1:100 dilution) and anti-APE1 (1:200 dilution) overnight. Cells were washed and incubated with the secondary antibodies Alexa Fluor 488 goat anti-mouse and Alexa Fluor 568 goat anti-rabbit (1:200) for 1 h at room temperature. Cells were finally washed and mounted with mounting medium containing DAPI (Abcam; ab104139). Images were captured using the BZ-X710 KEYENCE All-in-One Fluorescence Microscope.

### Immunohistochemistry (IHC)

Paraffin-embedded tissue slides were deparaffinized and rehydrated following standard protocols. Slides were then immersed for 20 min in boiling citrate buffer for antigen retrieval. Anti-APE1, anti-YAP1 and IHC Select Immunoperoxidase Secondary Detection System (DAB500, Millipore sigma) were utilized for staining following manufacturer’s instructions. IHC results were evaluated for intensity and frequency of the staining. The intensity of staining was graded as 0 (negative), 1 (weak), 2 (moderate) and 3 (strong). The frequency was graded from 0 to 4 by the percentage of positive cells as follows: grade 0, < 3%; grade 1, 3–25%; grade 2, 25–50%; grade 3, 50–75%; grade 4, > 75%. The index score was the product of multiplication of the intensity and frequency grades, which was then classified into a 4-point scale: index score 0 = product of 0, index score 1 = products 1 and 2, index score 2 = products 3 and 4, index score 3 = products 6–12.

### Animal experiments

All animal studies were conducted using protocols approved by the Institutional Animal Care and Use committee of the University of Miami (UM-20–110). The pL2-IL1β transgenic mice are a kind gift from Dr. Timothy Wang (Columbia University). It is a model of chronic esophageal inflammation that develops BE and EAC, as previously described [28]. The Mice were divided into 2 groups, untreated and bile salt, each containing 8 mice. The treated group received drinking water containing 0.3% deoxycholic acid (DCA) at neutral pH at the age of three months. After 7 months of continuous DCA administration, the mice were sacrificed and subjected to histological analysis of the squama-columnar junctions at the gastro-esophageal junctions.

De-identified patient-derived xenografts (PDXs) from human gastro-esophageal junction were generated according to our previously described platform [29]. PDX GTR0165 was used in the experiment. Samples were cut to a uniform size and implanted subcutaneously into bilateral flanks. Tumors were measured every other day until tumor volume reached approximately 150 mm^3^. The mice were then randomized into 2 groups, untreated and E3330, each containing 5 mice. E3330 was delivered via oral gavage (OG) at a dose of 20 mg/kg every weekday for 4 weeks. E3330 was dissolved in Cremophor: EtOH (1:1) (Cremophor was purchased from Sigma, Cat # C5135) for stock solution generation and diluted in PBS before injection. Tumor growth was determined by measuring the width and length of the tumors with an electronic caliper every 3 days. Body weights were measured every 7 days to monitor for drug toxicity. Tumor volume was calculated using the following formula: tumor volume (mm^3^) = 1/2 (W)^2^ x (L). At the experimental treatment endpoint of 4 weeks, mice were followed for survival. These mice were sacrificed once tumors reached 1000 mm^3^. A Kaplan–Meier survival estimate was performed with a log-rank calculation to determine statistical significance.

### Statistical analysis

All the statistical analyses were performed using GraphPad Prism, version 8.0 (GraphPad Software). Differences were analyzed by student’s t-test or one-way ANOVA followed by the Bonferroni post-hoc test. Results were reported as mean ± SD. *P* value < 0.05 was considered statistically significant. **, *p* < 0.05. ***, *p* < 0.01. N.S., no significance.

## Results

### APE1 regulates YAP1 expression and transcriptional activity in BE and EAC cell lines

**Fig. 1:**
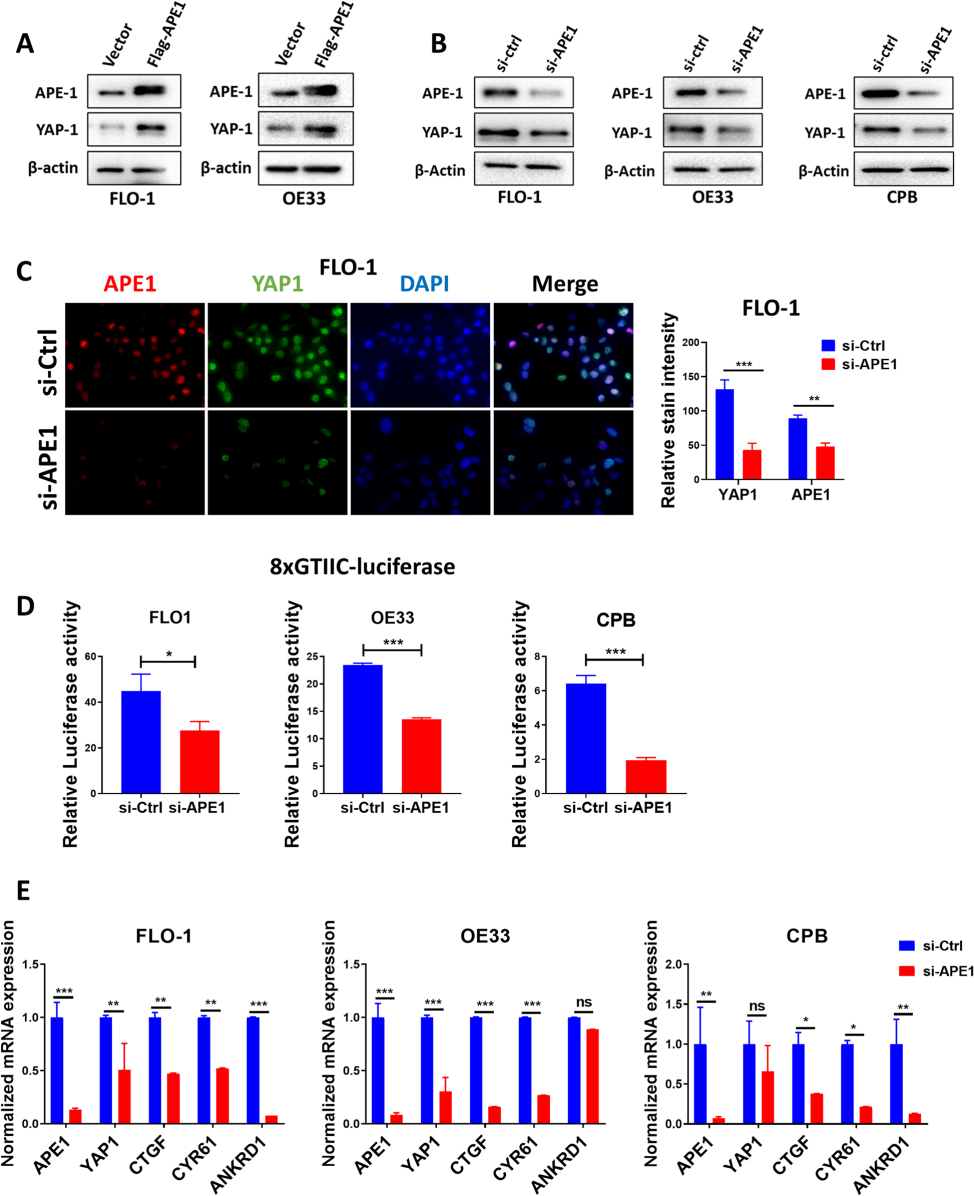
APE1 regulates YAP1 expression and transcriptional activity in BE and EAC cell lines. **A** FLO-1 (left) and OE33 (right) cells were transfected with APE1-wild-type overexpression plasmid and analyzed for APE1 and YAP1 expression by Western blot. β-actin was used as a loading control. **B** Western blot was used to analyze APE1 and YAP1 expression following transient APE1 knockdown (si-APE1) in FLO-1 (left), OE33 (middle) and CPB (right) cells. β-actin was used as a loading control. **C** Representative immunofluorescent staining images of APE1 (red) and YAP1 (green) in FLO-1 cells showing downregulation of nuclear YAP1 expression with or without APE1 knockdown. Quantification of stain intensity by ImageJ is also reported. DAPI (blue) was used for nuclear staining. **D** 8xCTIIC luciferase reporter assay was used to measure YAP1 transcriptional activity in FLO-1, OE33 and CPB cells with or without APE1 knockdown. The luciferase reporter activity values were normalized to Renilla expression levels. **E** qRT-PCR analyses of YAP1 downstream target genes, CTGF, CYR61 and ANKRD1 in FLO-1, OE33 and CPB cells with or without APE1 knockdown. Values are represented as mean ± SEM of three independent experiments. * *P* < 0.05; ** *P* < 0.01; *** *P* < 0.001, ns: no significance

We have previously reported that APE1 is induced by ABS in BE and EAC [4, 12]. Limited studies investigated the role of YAP1 and its regulation in EAC. We detected high levels of YAP1 and APE1 in BE and EAC cell lines (Suppl. Figure 1A). To determine if APE1 could regulate YAP1, we overexpressed or transiently knocked down APE1 in BE and EAC cells. Western blot analysis showed an increase in YAP1 protein level in FLO-1 and OE33 cells, following APE1 overexpression (Fig. 1A). Conversely, APE1 knockdown decreased YAP1 expression in FLO-1, OE33, and CPB cells, as compared to scrambled control (Fig. 1B). Immunofluorescence staining showed nuclear immunostaining pattern of YAP1 and APE1 in FLO-1 cells and demonstrated a decreased nuclear staining of YAP1 upon APE1 knockdown as compared to control cells (Fig. 1C). We then assessed the effect of APE1 knockdown on the YAP1 transcriptional activity using the 8xGTIIC luciferase reporter. APE1 knockdown significantly reduced YAP1 transcriptional activity in FLO-1, OE33, and CPB cells when compared to scrambled control (Fig. 1D). Concordant with these findings, we detected a significant decrease in the expression levels of YAP1 target genes, CTGF, CYR61 and ANKRD1, in APE1 knockdown cells as compared to control cells (Fig. 1E). Taken together, these results confirm a role of APE1 in regulating YAP1 protein level and transcription activity.

### ABS exposure induces YAP1 activation

**Fig. 2:**
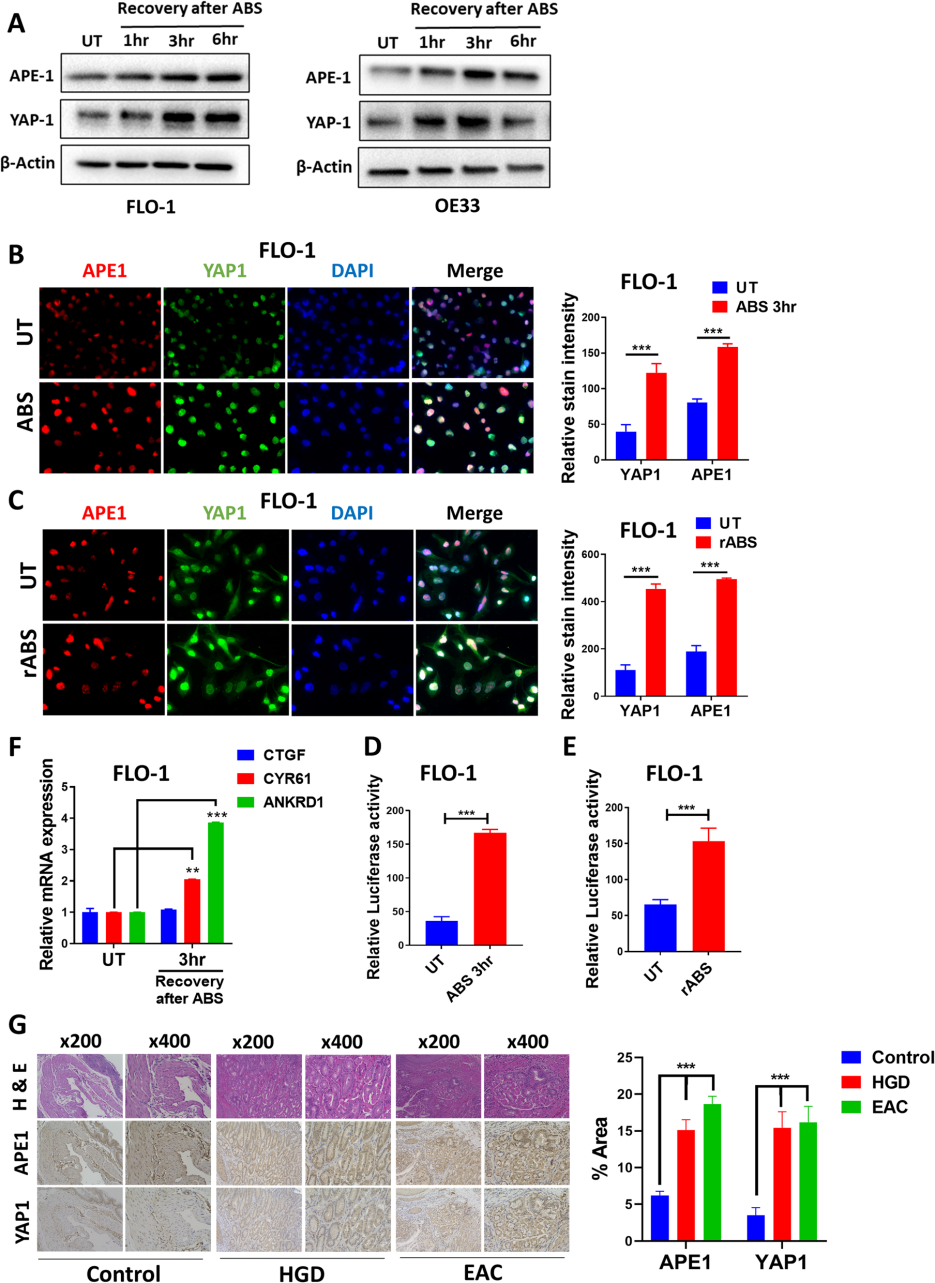
ABS induces YAP1 expression, promotes its nuclear accumulation and facilitates its transcriptional activity. **A** Western blot analysis of APE1 and YAP1 levels in FLO-1 and OE33 cells. Cells were exposed to 200 µM ABS for 20 min followed by recovery in complete media for the indicated time points. β-actin was used as a loading control. **B** Representative immunofluorescent staining images of APE1 (red) and YAP1 (green) in FLO-1 cells exposed to ABS (200 µM, pH 4, 20 min) followed by 3 h recovery versus control untreated cells. DAPI (blue) was used for nuclear staining. Quantification of stain intensity by ImageJ is also reported. **C** Representative immunofluorescent staining images of APE1 (red) and YAP1 (green) in FLO-1 cells repeatedly exposed to ABS (200 µM, pH 5.5, 20 min per day for 14 days) versus control untreated cells. DAPI (blue) was used for nuclear staining. Quantification of stain intensity by ImageJ is also reported. **D-E** YAP1 transcriptional activity was measured using 8xCTIIC luciferase reporter assay in FLO-1 cells exposed or not to ABS treatment (200 µM, pH 4, 20 min) followed by 3 h recovery (**D**) or FLO-1 cells repeatedly exposed to ABS (200 µM, pH 5.5, 20 min per day for 14 days) versus control untreated cells (**E**). The luciferase reporter activity values were normalized to Renilla expression levels. **F** qRT-PCR analyses of YAP1 downstream target genes, CTGF, CYR61 and ANKRD1 in FLO-1 cells exposed to ABS (200 µM, pH 4, 20 min) followed by 3 h recovery versus control untreated cells. Values are represented as mean ± SEM of three independent experiments. * *P* < 0.05; ** *P* < 0.01; *** *P* < 0.001. **G** Representative immunohistochemistry staining images of APE1 and YAP1 in L2-IL-1β transgenic mouse tissues with high-grade dysplasia (HGD) and esophageal adenocarcinoma (EAC) lesions (Untreated: 8mice; HGD and EAC: 8mice each). Quantification of stain intensity by ImageJ is shown

To determine if YAP1 is induced under reflux conditions, we treated cells with an ABS cocktail to mimic GERD episodes in patients. Cells were either transiently exposed to ABS (200 µM, pH 4.0) for 20 min followed by recovery in complete media for 1, 3, and 6 h, or repeatedly exposed to ABS (rABS; 200 µM, pH 5.5) for 20 min per day for 14 days. Western blot analysis showed a simultaneous increase in the protein levels of APE1 and YAP1, following transient (Fig. 2A, Suppl. Figure 1B) or repeated (Suppl. Figure 1C) exposure to ABS. Immunofluorescence staining demonstrated increased nuclear immunostaining of APE1 and YAP1 following transient or repeated ABS exposure in FLO-1 (Fig. 2B and C) and OE33 cells (Suppl. Figure 1D). Using the 8xGTIIC luciferase reporter assay, as a measure of YAP1 transcription activity, we detected a significant increase in YAP1 transcriptional activity in cells treated with ABS (*P* < 0.01), as compared with control cells (Fig. 2D and E, Suppl. Figure 2A and B). qRT-PCR analysis demonstrated the significant increase in the mRNA levels of YAP1 target genes, CYR61 and ANKRD1, following exposure to ABS (Fig. 2F, Suppl. Figure 2C, D and E). To validate our findings in vivo, we used the L2-IL-1β transgenic mouse model of BE/EAC [28]. These mice developed neoplastic lesions including high-grade dysplasia (HGD) or EAC lesions at the gastro-esophageal junction after 7 months of DCA exposure (Fig. 2G). IHC staining demonstrated high nuclear expression of APE1 and YAP1 in the neoplastic glandular areas at the gastro-esophageal junctions (Fig. 2G). Taken together, these results suggest up-regulation and activation of YAP1 in response to reflux conditions (ABS).

### APE1 mediates ABS-induced YAP1 activation

**Fig. 3:**
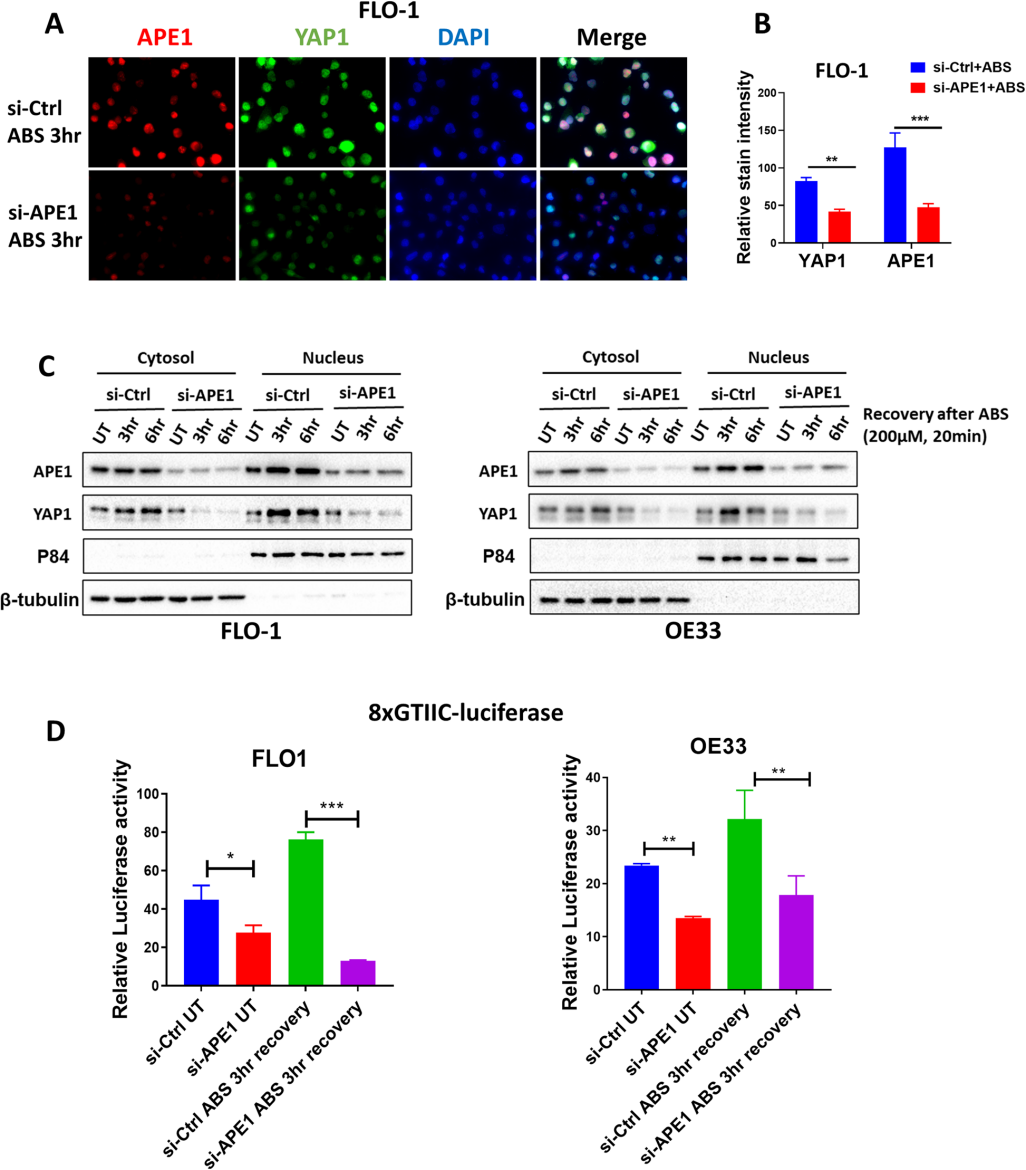
APE1 mediates ABS-induced YAP1 activation. **A** Representative immunofluorescence images of APE1 (red) and YAP1 (green) in FLO-1 cells exposed to ABS (200 µM, pH 4, 20 min) followed by 3 h recovery with or without APE1 knockdown. DAPI (blue) was used for nuclear staining. **B** Quantification of immunofluorescence stain intensity using ImageJ. **C** Cytosolic and nuclear fractions from FLO-1 (left) and OE33 (right) cells transfected with si-Ctrl or si-APE1 and treated with ABS (200 µM, pH 4, 20 min) followed by recovery in complete media for indicated timepoints were evaluated by western blot for the levels of APE1 and YAP1. β-tubulin and p84 were used as a loading control for cytosolic and nuclear fractions, respectively. **D** 8xCTIIC luciferase reporter assay was used to measure YAP1 transcriptional activity in FLO-1 and OE33 cells transfected with si-Ctrl or si-APE1 and exposed or not to ABS treatment (200 µM, pH 4, 20 min) followed by 3 h recovery. The luciferase reporter activity values were normalized to Renilla expression levels. Values are represented as mean ± SEM of three independent experiments. * *P* < 0.05; ** *P* < 0.01; *** *P* < 0.001

Our previous studies identified APE1 as an important player in protecting esophageal cancer cells from oxidative stress in conditions of ABS exposure [4, 11, 12]. We, therefore, hypothesized that APE1 could play a role in mediating ABS-induced YAP1 activation. Immunofluorescence and western blot analysis of nuclear and cytosolic fractions demonstrated an increase in nuclear APE1 and YAP1 expression in EAC cells following ABS exposure. APE1 knockdown inhibited the ABS-induced nuclear accumulation of YAP1 in these cells (Fig. 3A-C). Moreover, APE1 silencing abrogated ABS-enhanced YAP1 transcriptional activity (Fig. 3D). Collectively, these results indicate APE1-dependent activation of YAP1 under reflux conditions (ABS).

### ABS-induced YAP1 activation depends on APE1 redox activity

**Fig. 4:**
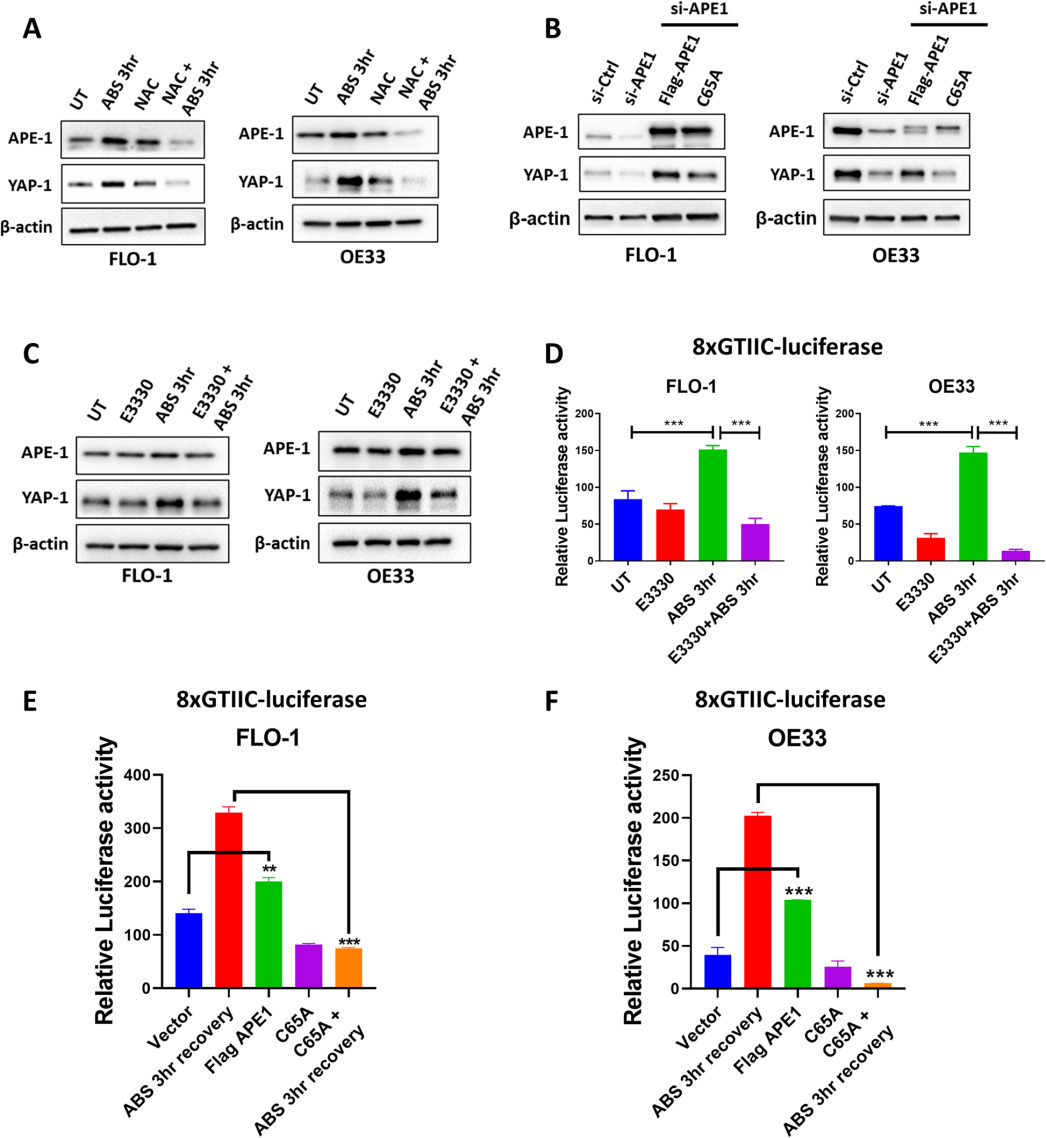
ABS-induced YAP1 activation depends on APE1 redox activity. **A** FLO-1 and OE33 cells were pre-treated or not with NAC (200 µM, 2 h) then exposed to ABS (200 µM, pH 4, 20 min) and allowed to recover for 3 h in complete media. Western blot analysis was used to evaluate APE1 and YAP1 protein levels. β-actin was used as a loading control. **B** FLO-1 and OE33 cells with transient knockdown of APE1 using si-APE1 were reconstituted with wild type APE1 (flag APE1) or APE1 redox mutant (C65A). Western blot was used to analyze the protein levels of APE1 and YAP1. β-actin was used as a loading control. **C** FLO-1 and OE33 cells were pre-treated or not with APE1 redox inhibitor E3330 (100 µM, overnight) then exposed to ABS (200 µM, pH 4, 20 min) and allowed to recover for 3 h in complete media with or without E3330. Western blot analysis was used to evaluate APE1 and YAP1 protein levels. β-actin was used as a loading control. **D** FLO-1 and OE33 cells were transfected with 8xCTIIC luciferase reporter and Renilla. Cells were then pre-treated or not with APE1 redox inhibitor E3330 (100 µM, overnight) then exposed to ABS (200 µM, pH 4, 20 min) and allowed to recover for 3 h in complete media with or without E3330. The relative 8xCTIIC luciferase reporter activity was measured and reported as mean ± SEM. * *P* < 0.05; ** *P* < 0.01; *** *P* < 0.001. **E–F** 8xCTIIC luciferase reporter assay was used to measure YAP1 transcriptional activity in FLO-1 (**E**) and OE33 (**F**) cells transfected or not with wild type APE1 (flag APE1) or APE1 redox mutant (C65A) with or without exposure to ABS (200 µM, pH 4, 20 min followed by 3 h recovery). The luciferase reporter activity values were normalized to Renilla expression levels. Values are represented as mean ± SEM. * *P* < 0.05; ** *P* < 0.01; *** *P* < 0.001

**Fig. 5:**
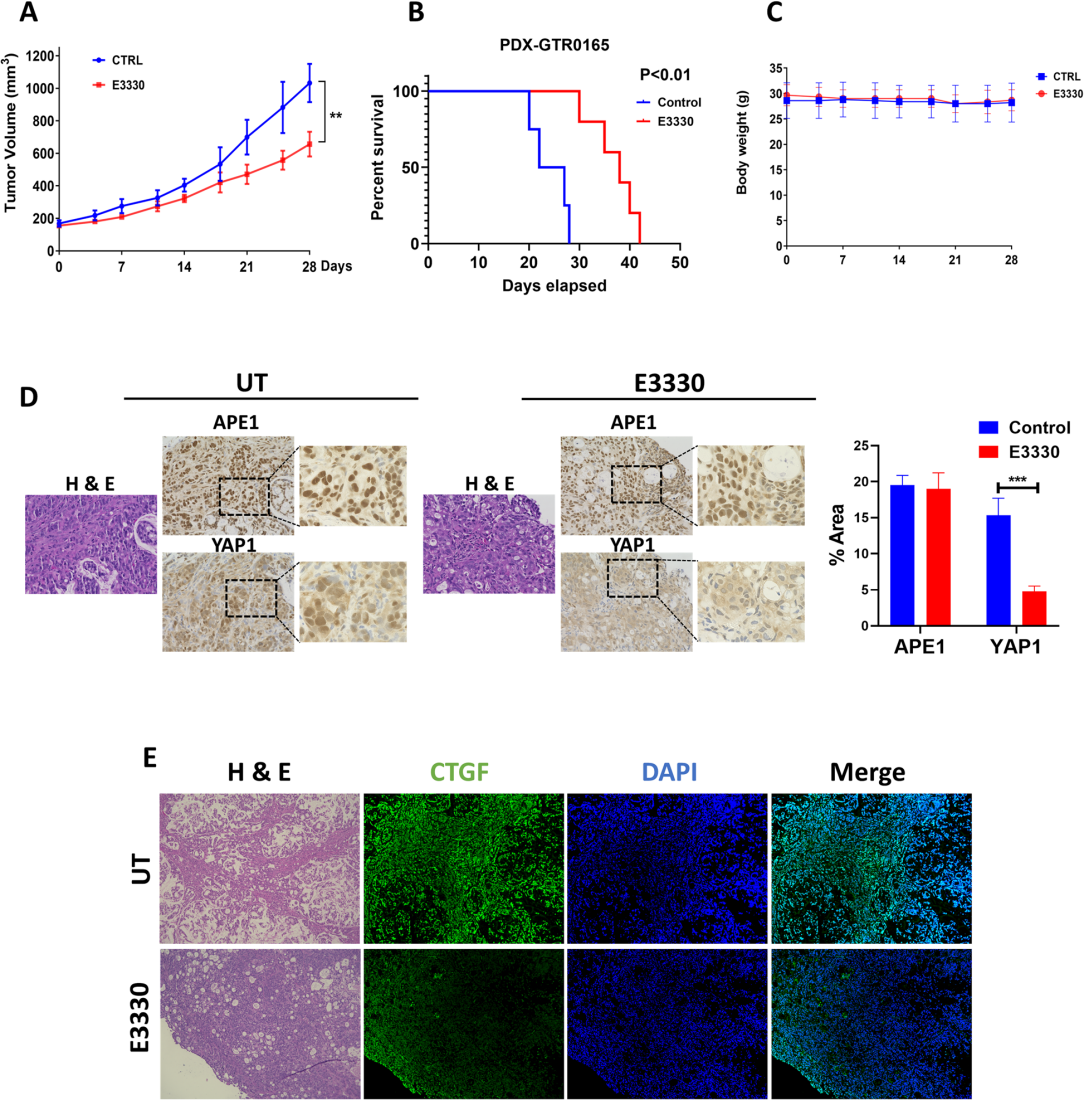
E3330 reduces PDX tumor growth and YAP1 activation. **A** Average tumor volume of PDX with or without 20 mg/kg E3330 treatment for 28 days (Control: 5mice; E3330: 5mice). **B** Kaplan–Meier survival curve for PDX following treatment endpoint. **C** Average weight of control and E3330 treated PDXs over 28 days of treatment. **D** Representative immunohistochemistry staining images of APE1 and YAP1 in PDX mouse tissues with or without E3330 treatment. Quantification of immunofluorescence intensity by ImageJ is also shown. **E** Representative immunofluorescence images of CTGF (green) in PDX mouse tissues with or without E3330 treatment. DAPI (blue) was used for nuclear staining

We first confirmed that oxidative stress mediates YAP1 induction using the ROS scavenger N-acetylcysteine (NAC). Western blot analysis demonstrated that NAC treatment inhibited YAP1 activation following exposure to ABS (Fig. 4A). To detect whether YAP1 regulation depends on APE1 redox activity, we reconstituted wild-type APE1 (flag-APE1) or APE1-redox-deficient-mutant (C65A) in APE1 knockdown EAC cells. Results indicated that wild-type APE1 and not the redox-deficient-mutant was able to induce YAP1 protein levels (Fig. 4B). To determine the role of APE1 redox function in ABS-induced YAP1 activation, we treated EAC cells with APE1 redox inhibitor, E3330, alone or in combination with ABS. Results showed that E3330 suppressed ABS-mediated increase in YAP1 protein level (Fig. 4C) and abrogated ABS-induced YAP1 transcriptional activity (Fig. 4D). We further confirmed these results by measuring YAP1 transcriptional activity under ABS conditions in control, APE1-redox-deficient-mutant (C65A) or wild-type APE1 (flag-APE1) transfected EAC cells. Results showed that APE1-redox-deficient-mutant failed to induce YAP1 transcriptional activity in response to ABS (Fig. 4E and F). To validate these findings in vivo, we used a PDX mouse model from human gastro-esophageal junction. Treatment with 20 mg/kg E3330 significantly inhibited PDX tumor volume (Fig. 5A, *P* < 0.01) and enhanced PDX mice survival (Fig. 5B, *P* < 0.01) compared with control tumors. E3330 treatment had no significant effect on mice weight suggesting limited toxicity (Fig. 5C). IHC staining for APE1 and YAP1 demonstrated downregulation of YAP1 nuclear expression in PDX mouse tissues treated with E3330 as compared to untreated control tissues (Fig. 5D). Concordant with these findings, immunofluorescence staining showed decreased expression of YAP1 target gene, CTGF, in E3330 treated PDXs (Fig. 5E). Taken together, these results indicate that reflux conditions promote YAP1 activation in an APE1-redox-dependent manner.

### APE1 regulates YAP1 stability through β-TrCP ubiquitinase

**Fig. 6:**
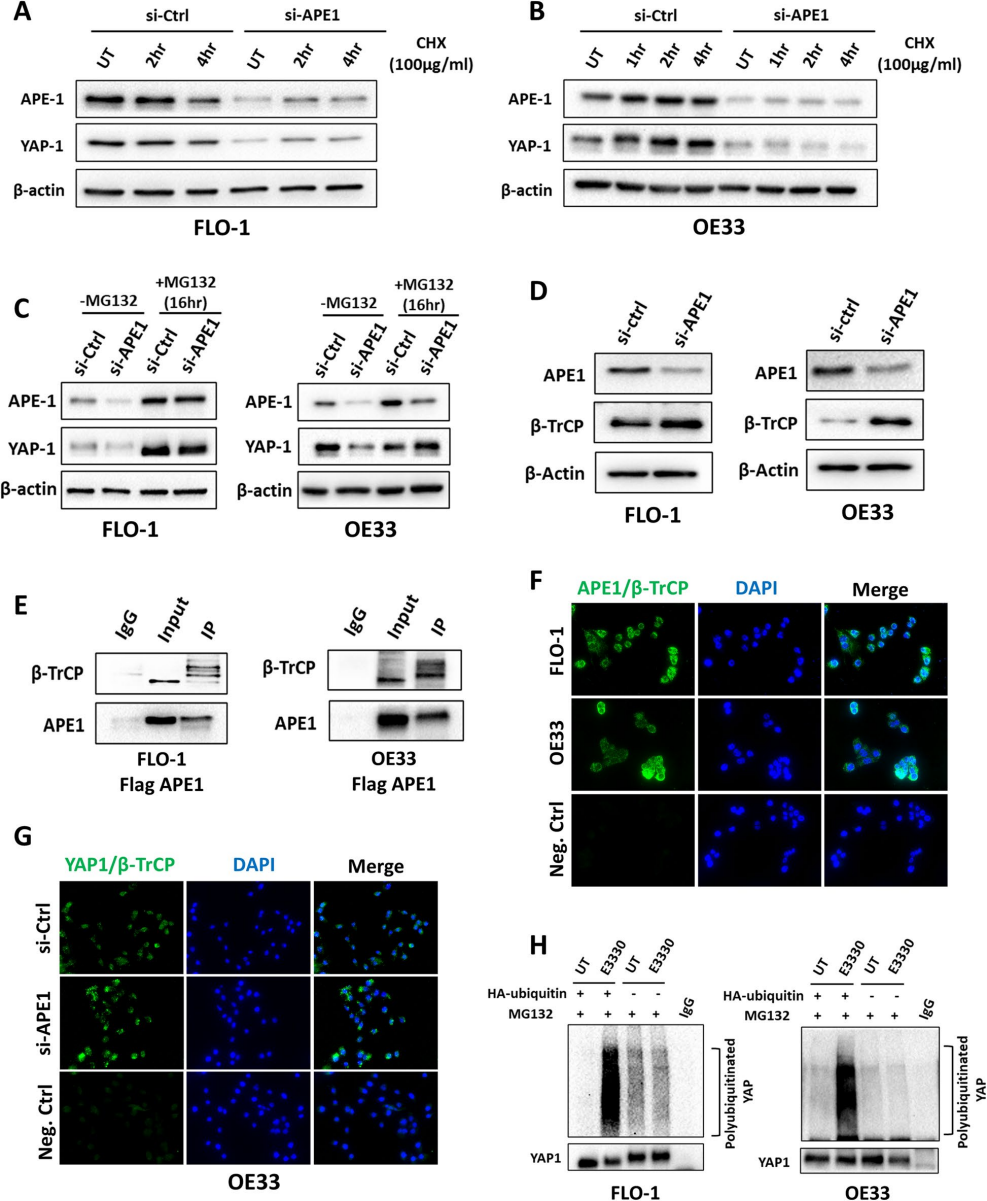
APE1 regulates YAP1 stability through β-TrCP ubiquitinase. **A-B** FLO-1 (**A**) and OE33 (**B**) cells with or without APE1 transient knockdown were treated with cycloheximide (CHX, 100 µg/mL) for the indicated timepoints. The levels of APE1 and YAP1 were determined using western blot. β-actin was used as a loading control. **C** FLO-1 and OE33 cells with or without APE1 transient knockdown were treated or not with MG132 (10 µM) for 16 h. The levels of APE1 and YAP1 were evaluated using western blot. β-actin was used as a loading control. **D** Western blot was used to analyze APE1 and β-TrCP expression following transient APE1 knockdown (si-APE1) in FLO-1 and OE33 cells. β-actin was used as a loading control. **E** FLO-1 and OE33 cells were transfected with wild type APE1 (flag APE1), immunoprecipitated with flag antibody and immunoblotted with β-TrCP and APE1 antibodies. **F-G** Representative images of proximity ligation assay for APE1 and β-TrCP in FLO-1 and OE33 cells (**F**) and YAP1 and β-TrCP in OE33 cells with and without APE1 knockdown (**G**). DAPI (blue) was used for nuclear staining. **H** In vitro ubiquitination assay in FLO-1 and OE33 cells transfected with HA-ubiquitin plasmid then treated or not with APE1 redox inhibitor E3330 (100 µM, overnight) followed by MG132 treatment (10 µM for 6 h). YAP1 antibody was used for immunoprecipitation and samples were then blotted with anti-ubiquitin and anti-YAP1

Because changes in mRNA expression levels of YAP1 were not significant across EAC cell lines (Suppl. Figure 3), we hypothesized that APE1 regulates YAP1 through a post-transcriptional mechanism at the protein level. To validate this assumption, we performed cycloheximide (CHX) chase assay in FLO-1 and OE33 cells with or without APE1 silencing. We found that YAP1 protein degraded much faster in APE1 knockdown cells, as compared to control cells (Fig. 6A and B). We further confirmed these results by treating control and APE1 knockdown FLO-1 and OE33 cells with the proteasome inhibitor, MG132. We found that treatment with MG132 restored APE1 and YAP1 protein expression when compared to untreated group (Fig. 6C).

A major regulatory process of YAP1 is ubiquitination by the SCFβ-TRCP E3 ubiquitin ligase and subsequent degradation [16]. We, therefore, investigated if APE1 regulates YAP1 stability via β-TRCP. Indeed, western blot results indicated that APE1 knockdown upregulated β-TRCP protein levels in FLO-1 and OE33 cells, as compared to scrambled control cells (Fig. 6D). To study this further, we performed immunoprecipitation assay (IP). The results demonstrated a novel protein interaction between β-TRCP and APE1 in FLO-1 and OE33 cells (Fig. 6E). In line with these results, proximity ligation assay data confirmed the close proximity of β-TRCP and APE1 proteins in both FLO-1 and OE33 cells, as compared to negative control (Fig. 6F). Knockdown of APE1 increased YAP1-β-TRCP interaction in OE33 cells (Fig. 6G). Importantly, in vitro ubiquitination assay showed that APE1 redox inhibition (E3330) induced YAP1 polyubiquitination when compared to untreated group (Fig. 6H). Taken together, these data indicate that APE1 mediates YAP1 protein stability through interaction with β-TRCP and possibly interfering with YAP1 binding to its degradation complex.

### ABS enhances sphere formation and induce YAP1 through APE1

**Fig. 7:**
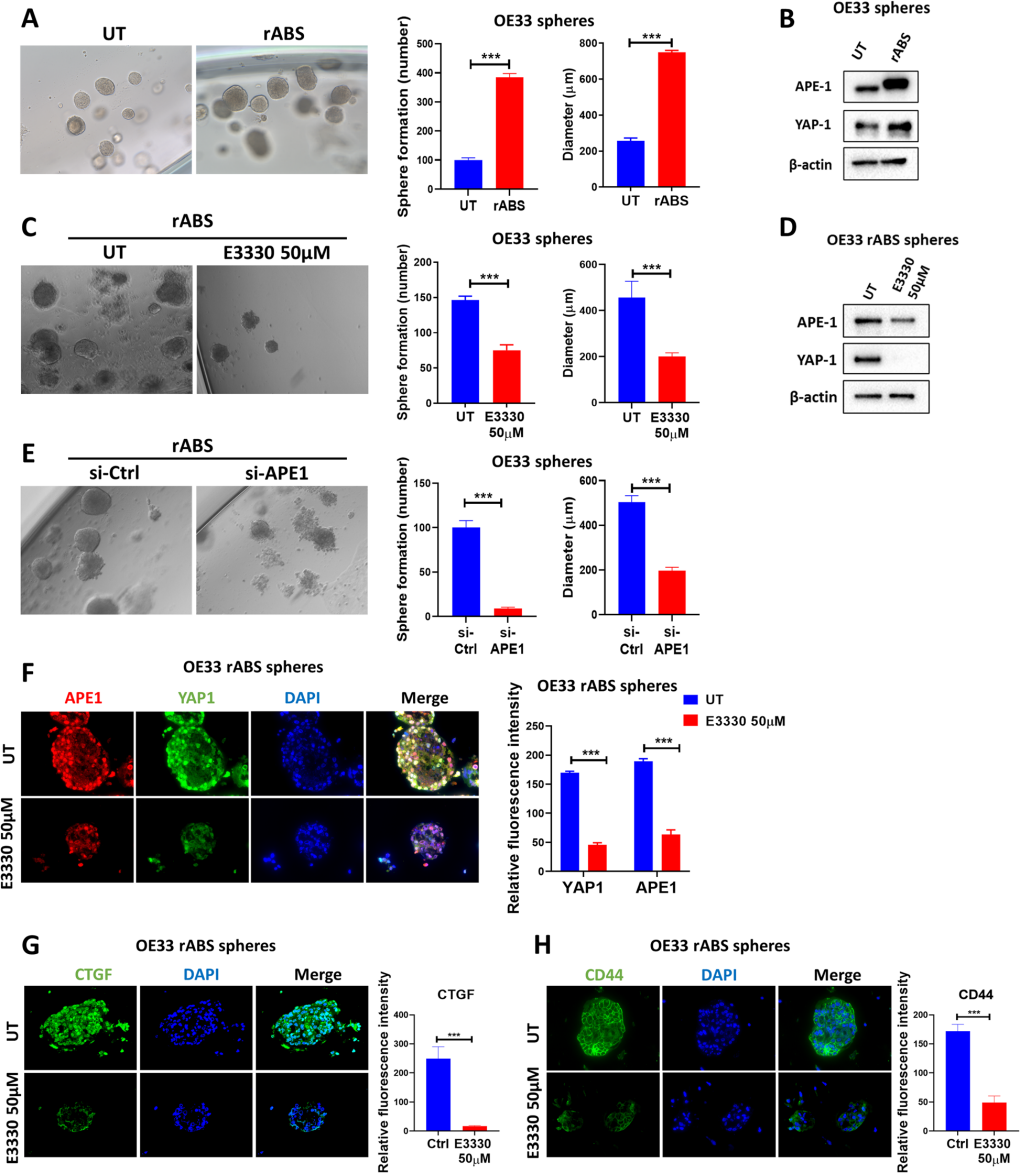
ABS enhances sphere formation and induces YAP1 through APE1. **A** Representative bright field images of OE33 spheres derived from control cells (UT) or cells repeatedly exposed to ABS (200uM, pH5.5) for 20 min per day for 14 days (rABS). Quantification of the number and diameter of spheres is also presented. **B** Western blot analysis of APE1 and YAP1 in OE33-derived spheres with or without repeated ABS. **C** Representative bright field images of spheres derived from repeated-ABS-treated OE33 cells with or without E3330 (50 µM) treatment. Quantification of the number and diameter of spheres is also presented. **D** Western blot analysis of APE1 and YAP1 in spheres derived from repeated-ABS-treated OE33 cells with or without E3330 (50 µM) treatment. **E** Representative bright field images of spheres derived from repeated-ABS-treated OE33 cells with or without transient APE1 knockdown. Quantification of the number and diameter of spheres is also presented. **F–H** Representative immunofluorescence images of spheres derived from repeated-ABS-treated OE33 cells with or without E3330 (50 µM) treatment showing APE1 (red) and YAP1 (green) staining in (**F**), CTGF (green) in (**G**) and CD44 (green) in (**H**). DAPI (blue) was used for nuclear staining. Quantification of immunofluorescence intensity by ImageJ is also shown. Values are represented as mean ± SEM. * *P* < 0.05; ** *P* < 0.01; *** *P* < 0.001

YAP1 plays an integral role in self-renewal and maintenance of cancer stemness [19, 30]. To investigate if APE1-YAP1 promotes stem-like features in response to reflux conditions, we exposed OE33 cells to repeated ABS treatment (200uM, pH = 5.5), 20 min per day for 14 days, followed by 3D sphere formation assay. OE33 cells with repeated ABS exposure displayed enhanced sphere forming ability depicted in the increased number and size of spheres, as compared with untreated control (Fig. 7A). Western blot analysis showed that spheres derived from cells exposed to repeated ABS treatment had higher levels of APE1 and YAP1 (Fig. 7B), confirming activation of these markers in response to reflux conditions. To determine if these phenotypic and molecular changes are mediated by APE1 through its redox function, we knocked down or treated repeated-ABS-OE33-derived spheres with 50 μM of the APE1 redox inhibitor, E3330. Silencing or treatment with E3330 significantly reduced the number and size of spheres compared with untreated control and inhibited YAP1 protein induction (Fig. 7C, D and E). Immunofluorescence staining demonstrated significant decrease in APE1, YAP1, CTGF and stem cell marker CD44 levels in spheres treated with E3330 (Fig. 7F, G and H). To further confirm these results, we transfected spheres derived from repeated-ABS-treated OE33 cells with APE1 si-RNA. Transient knockdown of APE1 led to a significant reduction in the number of spheres and the immunofluorescence stain intensity of YAP1, CTGF and CD44, as compared with control si-RNA (si-Ctrl) (Suppl. Figure 4A and B). Taken together, these results demonstrate an important role of APE1-YAP1 in promoting stem-like features in response to reflux conditions.

## Discussion

The incidence of esophageal adenocarcinoma continues to increase rapidly despite advances in treatment [31]. The etiology of EAC remains uncertain but has been linked to a variety of risk factors where gastroesophageal reflux disease (GERD) is considered a major risk factor for the development of BE and its progression to EAC [32]. Exposure to ABS, the mimicry of GERD episodes, was shown to induce overexpression of APE1 in BE and EAC cells as a mechanism of cell survival and homeostasis [4, 12, 15]. The molecular functions of APE1 in BE and EAC remain largely understudied. We report a novel function of APE1 in EAC tumorigenesis through regulation of YAP1 signaling.

YAP1 is a transcriptional co-activator that has important function in normal tissue homeostasis and regeneration. Deregulation and consecutive activation of YAP1 is involved in cancer initiation, progression, metastasis, and therapeutic resistance [16]. YAP1 has been shown to be upregulated in high-grade dysplasia and EAC [21], suggesting a role of this pathway in EAC tumorigenesis. However, the mechanism of YAP1 activation and regulation in reflux conditions and EAC remains to be determined. Our findings in this study provide multiple lines of evidence supporting the role of APE1 as an upstream activator of YAP1 in EAC. We consistently found that knockdown of APE1 remarkably diminished YAP1 activity and downstream signaling. Of note, exposure of esophageal cells to ABS resulted in elevated YAP1 activity in vitro and in vivo, suggesting a role of reflux conditions in YAP1 activation. Interestingly, we found that YAP1 activation under reflux conditions was APE1 dependent. APE1 was shown to mediate tumor progression through activation of various MAPK signaling cascade components such as STAT3, Src and ERK [8, 33]. Earlier reports have shown that MAPK signaling was associated with drug resistance and metastasis through direct activation of YAP1 [34, 35]. Therefore, it is also possible that MAPK may contribute to APE1-mediated activation of YAP1 induction in response to reflux, warranting further investigation.

APE1 has a unique redox activity, mediated by cysteine 65, required for maintaining the activity of redox-dependent transcription factors and several kinases [36]. Unlike the wild-type APE1, reconstitution with APE1 redox-deficient mutant (C65A) failed to induce YAP1 activation. In addition, treatment with APE1 redox specific inhibitor E3330 inhibited YAP1 induction. These results were recapitulated in vivo, using a PDX mouse model. Taken together, these novel results indicate the role of APE1 redox activity in YAP1 activation. It is important to mention that E3330 treatment reduced tumor volume in PDX mouse model but was not associated with tumor regression suggesting the limited efficacy of E3330 as a single agent and highlighting the need for investigating combinatorial strategies with other chemotherapeutic agents.

**Fig. 8:**
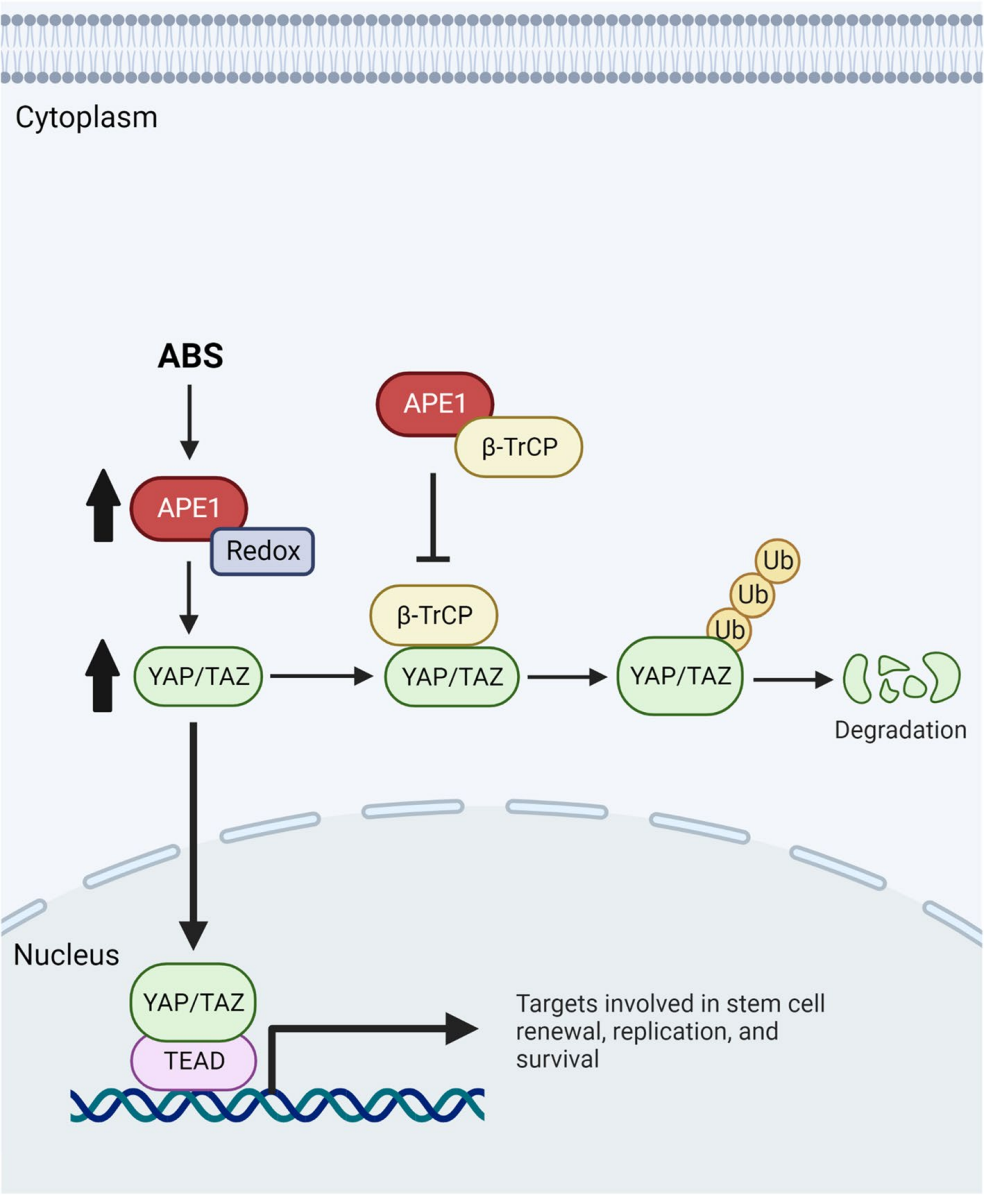
A schematic diagram illustrating the role of APE1 in activating and stabilizing YAP1. Exposure of cells to acidic bile salts (ABS) induce APE1-dependent increase in YAP1, leading to its activation and translocation into the nucleus. Mechanistically, APE1 stabilizes YAP1 by binding to β-TrCP thereby disrupting YAP1-β-TrCP interaction and preventing YAP1 polyubiquitination and degradation

Our results indicated that APE1 is required for maintaining YAP1 protein stability. It is known that YAP1 activity can be regulated by several different mechanisms. A major mechanism of YAP1 inactivation is mediated by proteosomal degradation through which phosphorylation of YAP1 at multiple sites results in the formation of a phosphodegron that primes YAP1 for ubiquitination by the SCFβ-TRCP E3 ubiquitin ligase and subsequent degradation [37–39]. Our results uncovered a direct interaction between APE1 and β-TRCP, suggesting that APE1 regulates YAP1 degradation. Indeed, the results suggest that YAP1 stability is maintained through APE1 redox activity, providing a plausible explanation for the observed increase in YAP1 protein and activity levels (Fig. 8).

YAP1 plays an integral role in self-renewal and cellular expansion [19, 30]. YAP1 is known to correlate with expansion of cancer stem cells and tumor progression through interaction with cancer-promoting pathways, such as the NOTCH, and WNT pathways. Recent studies have shown that YAP1 is highly expressed in liver cancer stem cells and its expression was positively correlated with the expression of stemness markers (NANOG, OCT-3/4, and CD133) [40]. Moreover, induction of YAP1 by NOTCH can promote the expression of SOX9 and EpCAM stemness markers in liver cancer [41]. Studies have also found that YAP1 plays a crucial role in the maintenance and expansion of cancer stem cells in breast, lung, and prostate cancer [42, 43]. In esophageal cancer, YAP1 was shown to confer cancer stem cell properties by activating SOX9 via TEAD-mediated binding [22, 23]. Our current findings indicated that exposure to reflux conditions enhanced sphere-forming ability and induced YAP1 in an APE1 redox-dependent manner, suggesting an important role of APE1-YAP1 in promoting stem-like features in response to reflux conditions.

## Conclusions

In summary, we have identified APE1-redox function as a major regulator of YAP1 activity under reflux conditions. This APE1-YAP1 axis may be critical in promoting EAC initiation and progression. Our findings warrant further investigations for testing APE1 inhibitors for prevention or therapeutic intervention in EAC.

## Supplementary Information

**Additional file 1: Suppl. Figure 1.** YAP1 is expressed in BE and EAC cells and upregulated by ABS. A) Western blot analysis of APE1 and YAP1 protein levels in esophageal cell lines from Barrett’s (BART, BAR10-T, CPA), dysplastic Barrett’s (CPB) and EAC (FLO-1, OE33, OE19 and SK4). β-actin was used as a loading control. B) Western blot analysis of APE1 and YAP1 levels in SK4, BART and EpC2 cells. Cells were exposed to 100 or 200 µM ABS for 20 minutes followed by recovery in complete media for the indicated time points. β-actin was used as a loading control. C) Western blot analysis of APE1 and YAP1 protein levels in OE33 cells repeatedly treated with ABS (200uM, pH5.5, 20 minutes per day for 14 days). D) Representative immunofluorescent staining images of APE1 (red) and YAP1 (green) in OE33 cells repeatedly exposed to ABS (200 µM, pH 5.5, 20 minutes per day for 14 days) versus control untreated cells. DAPI (blue) was used for nuclear staining. **Suppl. Figure 2.** ABS promotes YAP1 transcriptional activity, and its induction is APE1 dependent. A-B) YAP1 transcriptional activity was measured using 8xCTIIC luciferase reporter assay in OE33 cells exposed or not to ABS treatment (200 µM, pH 4.4, 20 minutes) followed by 3 hours recovery (A) or OE33 cells repeatedly exposed to ABS (200 µM, pH 5.5, 20 minutes per day for 14 days) versus control untreated cells (B). The luciferase reporter activity values were normalized to Renilla expression levels. C-E) qRT-PCR analyses of YAP1 downstream target genes, CTGF, CYR61 and ANKRD1 in OE33 (C), SK4 (D) and BART (E) cells exposed to ABS (200 µM, pH 4.4, 20 minutes) followed by 3 hours recovery versus control untreated cells. Values are represented as mean ± SEM of three independent experiments. * *P*<0.05; ** *P*<0.01; *** *P*<0.001. **Suppl. Figure 3.** ABS has no significant change on mRNA expression levels of YAP1 across EAC cell lines. A-D) qRT-PCR analyses of YAP1 in FLO-1 (A), OE33 (B), SK4 (C) and BART (D) cells exposed to ABS (200 µM, pH 4, 20 minutes) followed by 3 hours recovery versus control untreated cells. Values are represented as mean ± SEM of three independent experiments. * *P*<0.05; ** *P*<0.01; *** *P*<0.001. **Suppl. Figure 4.** ABS induces YAP1 through APE1 in OE33 spheres. A) Representative immunofluorescence images of APE1 (red) and YAP1 (green) in spheres derived from repeated-ABS-treated OE33 cells with or without APE1 knockdown. Quantification of stain intensity by ImageJ is shown. Values are represented as mean ± SEM. * P<0.05; ** P<0.01; *** P<0.001. B) Representative immunofluorescence images of CTGF and CD44 (green) in spheres derived from repeated-ABS-treated OE33 cells with or without APE1 knockdown.

## Data Availability

All data generated or analyzed during this study are included in this published article and its supplementary information files.

## Abbreviations

ABS: Acidic bile salts
APE1: Apurinic/apyrimidinic endonuclease
REF1: Redox factor 1
YAP1: Yes-associated protein 1
BE: Barrett’s esophagus
DAPI: 4′, 6-Diamidino-2-phenylindole
DCA: Deoxycholic acid
EAC: Esophageal adenocarcinomas
GERD: Chronic gastroesophageal reflux disease
IHC: Immunohistochemistry
qRT-PCR: Quantitative real-time polymerase chain reaction
FBS: Fetal bovine serum
WB: Western blot
IF: Immunofluorescence
HGD: High-grade dysplasia
CHX: Cycloheximide
PLA: Proximity ligation assay
PDXs: Patient-derived xenografts
