# Supplementary material for: APE1 redox function is required for activation of Yes-associated protein 1 under reflux conditions in Barrett’s-associated esophageal adenocarcinomas
Source: J Exp Clin Cancer Res. 2022 Sep 1;41:264. doi: 10.1186/s13046-022-02472-5 (PMC9434868; doi:10.1186/s13046-022-02472-5)
Supplement: Supplementary file 1 — Additional file 1: Suppl. Figure 1. YAP1 is expressed in BE and EAC cells and upregulated by ABS. A) Western blot analysis of APE1 and YAP1 protein levels in esophageal cell lines from Barrett’s (BART, BAR10-T, CPA), dysplastic Barrett’s (CPB) and EAC (FLO-1, OE33, OE19 and SK4). β-actin was used as a loading control. B) Western blot analysis of APE1 and YAP1 levels in SK4, BART and EpC2 cells. Cells were exposed to 100 or 200 µM ABS for 20 minutes followed by recovery in complete media for the indicated time points. β-actin was used as a loading control. C) Western blot analysis of APE1 and YAP1 protein levels in OE33 cells repeatedly treated with ABS (200uM, pH5.5, 20 minutes per day for 14 days). D) Representative immunofluorescent staining images of APE1 (red) and YAP1 (green) in OE33 cells repeatedly exposed to ABS (200 µM, pH 5.5, 20 minutes per day for 14 days) versus control untreated cells. DAPI (blue) was used for nuclear staining. Suppl. Figure 2. ABS promotes YAP1 transcriptional activity, and its induction is APE1 dependent. A-B) YAP1 transcriptional activity was measured using 8xCTIIC luciferase reporter assay in OE33 cells exposed or not to ABS treatment (200 µM, pH 4.4, 20 minutes) followed by 3 hours recovery (A) or OE33 cells repeatedly exposed to ABS (200 µM, pH 5.5, 20 minutes per day for 14 days) versus control untreated cells (B). The luciferase reporter activity values were normalized to Renilla expression levels. C-E) qRT-PCR analyses of YAP1 downstream target genes, CTGF, CYR61 and ANKRD1 in OE33 (C), SK4 (D) and BART (E) cells exposed to ABS (200 µM, pH 4.4, 20 minutes) followed by 3 hours recovery versus control untreated cells. Values are represented as mean ± SEM of three independent experiments. * P<0.05; ** P<0.01; *** P<0.001. Suppl. Figure 3. ABS has no significant change on mRNA expression levels of YAP1 across EAC cell lines. A-D) qRT-PCR analyses of YAP1 in FLO-1 (A), OE33 (B), SK4 (C) and BART (D) cells exposed to ABS (200 µM, pH [file 13046_2022_2472_MOESM1_ESM.docx]

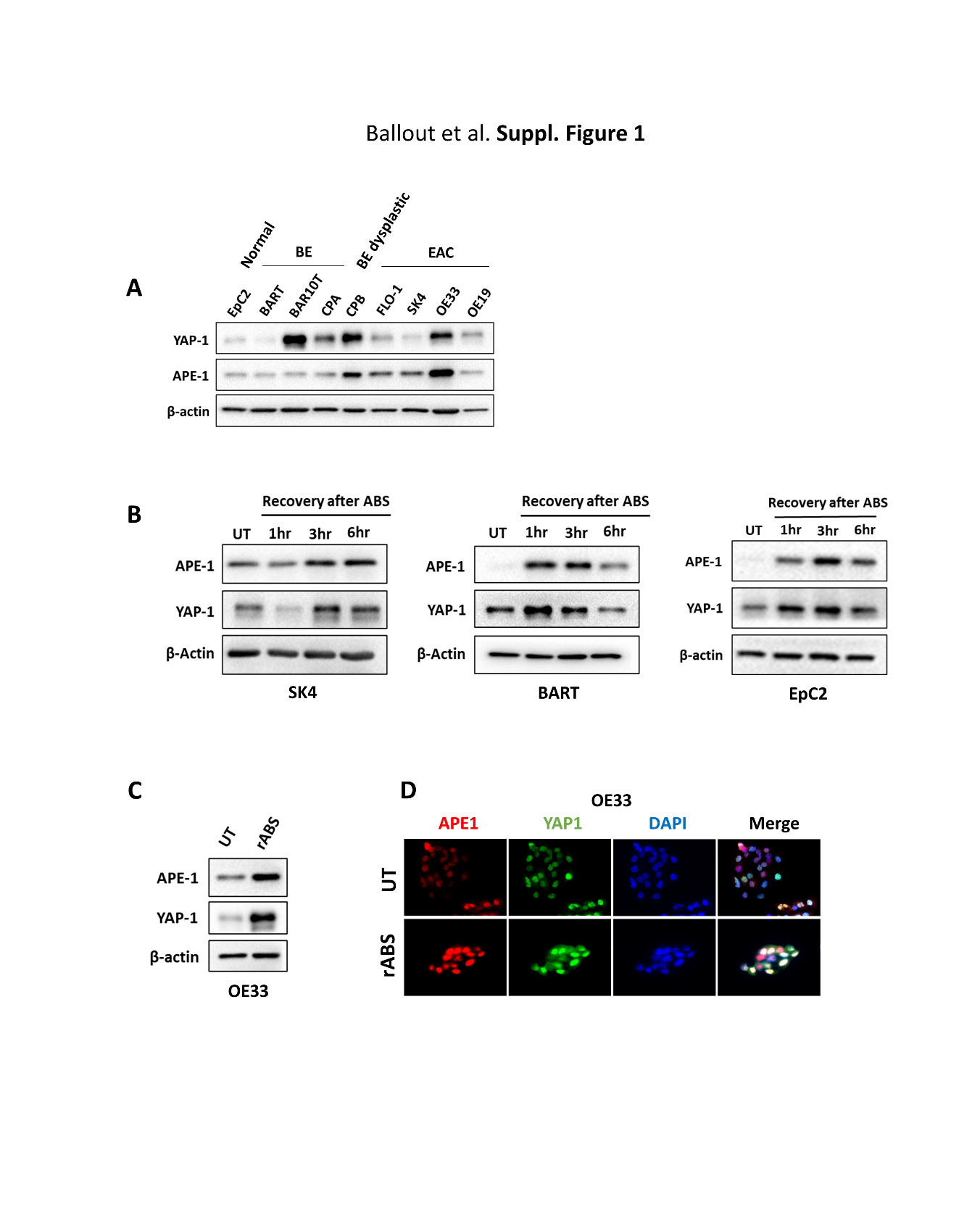


**Suppl. Figure 1.** YAP1 is expressed in BE and EAC cells and upregulated by ABS. **A)** Western blot analysis of APE1 and YAP1 protein levels in esophageal cell lines from Barrett’s (BART, BAR10-T, CPA), dysplastic Barrett’s (CPB) and EAC (FLO-1, OE33, OE19 and SK4). β-actin was used as a loading control. **B)** Western blot analysis of APE1 and YAP1 levels in SK4, BART and EpC2 cells. Cells were exposed to 100 or 200 µM ABS for 20 minutes followed by recovery in complete media for the indicated time points. β-actin was used as a loading control. **C)** Western blot analysis of APE1 and YAP1 protein levels in OE33 cells repeatedly treated with ABS (200uM, pH5.5, 20 minutes per day for 14 days). **D)** Representative immunofluorescent staining images of APE1 (red) and YAP1 (green) in OE33 cells repeatedly exposed to ABS (200 µM, pH 5.5, 20 minutes per day for 14 days) versus control untreated cells. DAPI (blue) was used for nuclear staining.


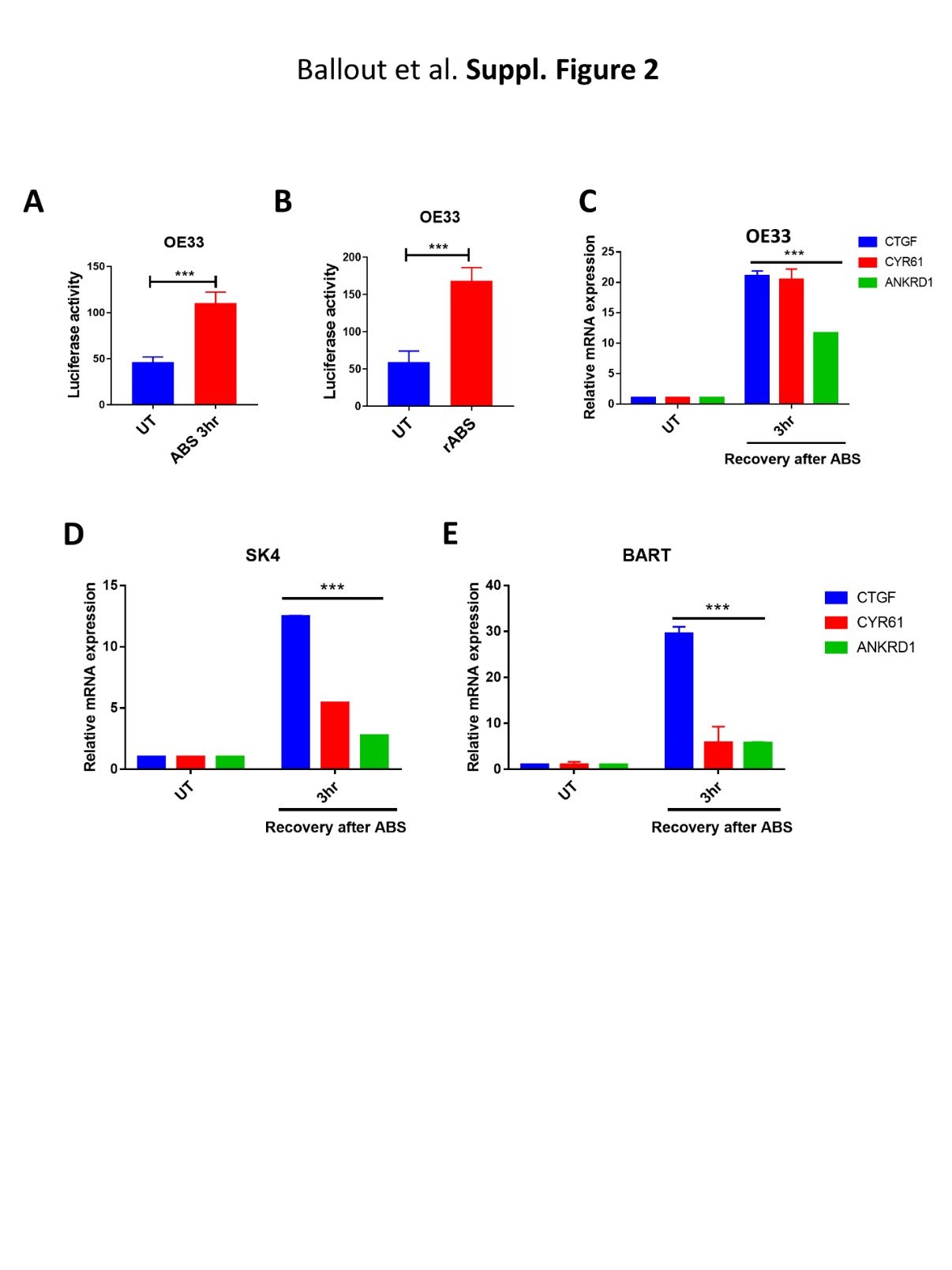


**Suppl. Figure 2.** ABS promotes YAP1 transcriptional activity, and its induction is APE1 dependent. **A-B)** YAP1 transcriptional activity was measured using 8xCTIIC luciferase reporter assay in OE33 cells exposed or not to ABS treatment (200 µM, pH 4.4, 20 minutes) followed by 3 hours recovery **(A)** or OE33 cells repeatedly exposed to ABS (200 µM, pH 5.5, 20 minutes per day for 14 days) versus control untreated cells **(B)**. The luciferase reporter activity values were normalized to Renilla expression levels. **C-E)** qRT-PCR analyses of YAP1 downstream target genes, CTGF, CYR61 and ANKRD1 in OE33 **(C)**, SK4 **(D)** and BART **(E)** cells exposed to ABS (200 µM, pH 4.4, 20 minutes) followed by 3 hours recovery versus control untreated cells. Values are represented as mean ± SEM of three independent experiments. * P<0.05; ** P<0.01; *** P<0.001.


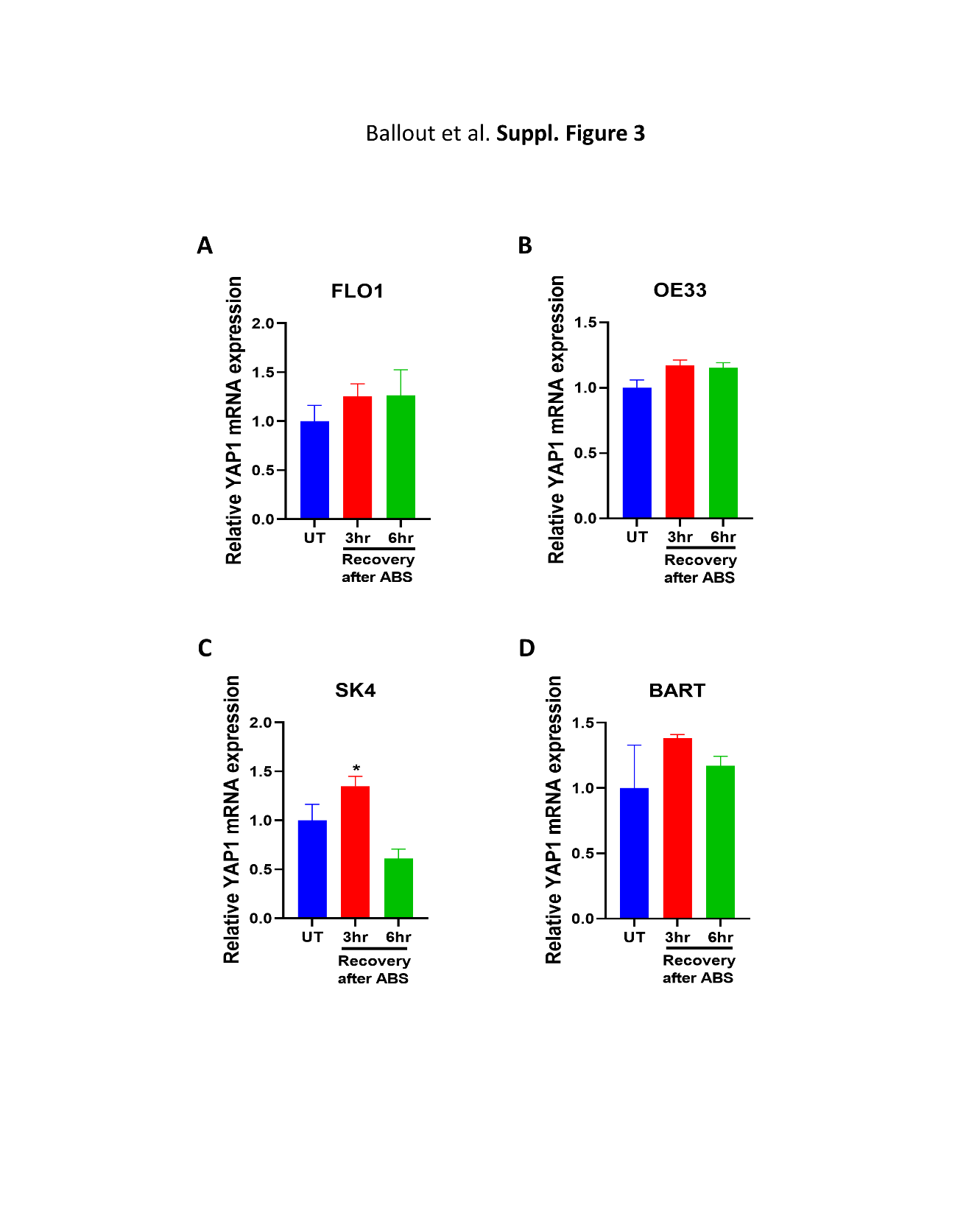


**Suppl. Figure 3.** ABS has no significant change on mRNA expression levels of YAP1 across EAC cell lines. A-D) qRT-PCR analyses of YAP1 in FLO-1 (A), OE33 (B), SK4 (C) and BART (D) cells exposed to ABS (200 µM, pH 4, 20 minutes) followed by 3 hours recovery versus control untreated cells. Values are represented as mean ± SEM of three independent experiments. * P<0.05; ** P<0.01; *** P<0.001.


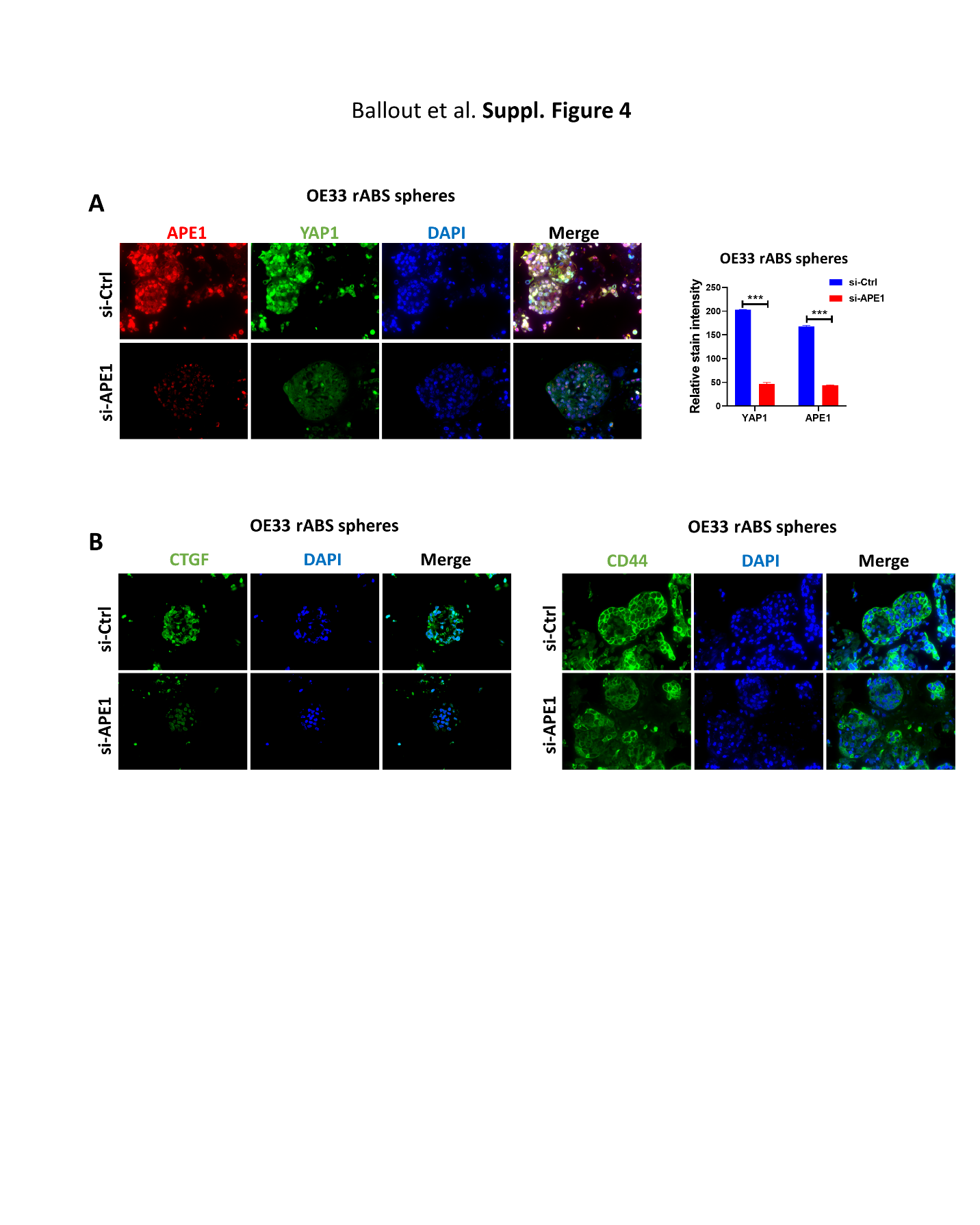


**Suppl. Figure 4.** ABS induces YAP1 through APE1 in OE33 spheres. **A)** Representative immunofluorescence images of APE1 (red) and YAP1 (green) in spheres derived from repeated-ABS-treated OE33 cells with or without APE1 knockdown. Quantification of stain intensity by ImageJ is shown. Values are represented as mean ± SEM. * P<0.05; ** P<0.01; *** P<0.001. **B)** Representative immunofluorescence images of CTGF and CD44 (green) in spheres derived from repeated-ABS-treated OE33 cells with or without APE1 knockdown.
